# AI-Based Algorithm to Detect Heart and Lung Disease From Acute Chest Computed Tomography Scans: Protocol for an Algorithm Development and Validation Study

**DOI:** 10.2196/77030

**Published:** 2025-09-19

**Authors:** Anne Sophie Overgaard Olesen, Kristina Miger, Silas Nyboe Ørting, Jens Petersen, Marleen de Bruijne, Mikael Ploug Boesen, Michael Brun Andersen, Johannes Grand, Jens Jakob Thune, Olav Wendelboe Nielsen

**Affiliations:** 1 Department of Cardiology Bispebjerg Hospital Copenhagen Denmark; 2 Department of Computer Science University of Copenhagen Copenhagen Denmark; 3 Department of Radiology Bispebjerg Hospital Copenhagen Denmark; 4 Department of Radiology Herlev Hospital Herlev Denmark; 5 Department of Cardiology Hvidovre Hospital Hvidovre Denmark; 6 Department of Clinical Medicine University of Copenhagen Copenhagen Denmark

**Keywords:** artificial intelligence, AI, computed tomography, machine learning, cardiac decompensation, dyspnea, acute care, diagnostic imaging

## Abstract

**Background:**

Dyspnea is a common cause of hospitalization, posing diagnostic challenges among older adult patients with multimorbid conditions. Chest computed tomography (CT) scans are increasingly used in patients with dyspnea and offer superior diagnostic accuracy over chest radiographs but face limited use due to a shortage of radiologists.

**Objective:**

This study aims to develop and validate artificial intelligence (AI) algorithms to enable automatic analysis of acute CT scans and provide immediate feedback on the likelihood of pneumonia, pulmonary embolism, and cardiac decompensation. This protocol will focus on cardiac decompensation.

**Methods:**

We designed a retrospective method development and validation study. This study has been approved by the Danish National Committee on Health Research Ethics (1575037). We extracted 4672 acute chest CT scans with corresponding radiological reports from the Copenhagen University Hospital–Bispebjerg and Frederiksberg, Denmark, from 2016 to 2021. The scans will be randomly split into training (2/3) and internal validation (1/3) sets. Development of the AI algorithm involves parameter tuning and feature selection using cross validation. Internal validation uses radiological reports as the ground truth, with algorithm-specific thresholds based on true positive and negative rates of 90% or greater for heart and lung diseases. The AI models will be validated in low-dose chest CT scans from consecutive patients admitted with acute dyspnea and in coronary CT angiography scans from patients with acute coronary syndrome.

**Results:**

As of August 2025, CT data extraction has been completed. Algorithm development, including image segmentation and natural language processing, is ongoing. However, for pulmonary congestion, the algorithm development has been completed. Internal and external validation are planned, with overall validation expected to conclude in 2025 and the final results to be available in 2026.

**Conclusions:**

The results are expected to enhance clinical decision-making by providing immediate, AI-driven insights from CT scans, which will be beneficial for both clinicians and patients.

**International Registered Report Identifier (IRRID):**

DERR1-10.2196/77030

## Introduction

### Background

Dyspnea accounts for up to 15% of all acute hospital admissions [1], and patients with dyspnea are often older adults and have multimorbid conditions. Correctly diagnosing the cause of dyspnea as either heart or pulmonary disease in emergency settings can be challenging, and 20% of the patients with acute heart failure are initially misdiagnosed [2]. Chest radiographs have long been the primary diagnostic tool for acute dyspnea [2]. However, cardiac decompensation and signs of pneumonia are often overlooked on chest radiographs, especially in older adult patients with chronic lung disease [3]. In addition, many patients with acute dyspnea cannot stand while undergoing chest radiographs, which further limits their diagnostic value.

Chest computed tomography (CT) scans offer the highest standard of care modality for patients with comorbid conditions and emergent conditions, such as pulmonary embolism, aortic dissection, pneumothorax, and chest trauma. Moreover, the diagnostic use of CT imaging for patients with dyspnea has increased [4]. CT scans allow for fast image acquisition and depict anatomy in cross section and 3 dimensions, providing superior information compared to conventional chest radiographs on diseases in the pulmonary parenchyma, heart, and vessels [5]. In our previous work, we identified 5 characteristic CT signs that were strongly associated with acute heart failure in patients with dyspnea [6] and later demonstrated that CT was 4 times more likely than chest radiography to detect cardiac decompensation in the emergency department [7]. Building on these findings, we now aim to enable automatic detection of cardiac decompensation on CT.

With an expected increase in older adults and patients with comorbid conditions admitted with dyspnea, there will be a greater need for accurate and fast diagnostic methods in the emergency departments. Over the course of the last 2 decades, there has been a worldwide increase in the number of CT scans [4]. In combination with a shortage of radiologists, this limits the broad use of chest CT imaging for patients with acute dyspnea in the emergency setting [8]. Automated diagnostic tools have the potential to enhance radiological efficiency by expediting the assessment and triage of patients presenting with acute dyspnea, thereby streamlining workflow in acute care settings. Conditions, such as cardiac decompensation, pneumonia, and pulmonary embolism, require timely diagnosis and intervention to improve clinical outcomes [1,2,9], making them appropriate targets for such models.

While the potential of artificial intelligence (AI) to support physicians in acute care is substantial, several challenges are particularly relevant for patients with dyspnea. Older adults and populations with comorbid conditions may introduce demographic bias and limit generalizability across settings, and variations in CT protocols, scanner types, and image quality can affect feature representation. Furthermore, interpretability is essential to ensure clinical applicability. In this protocol, we outline strategies to address these challenges by including diverse training data and multiple CT protocols and planning model outputs that are clinically interpretable with validation as a central component.

Previous AI studies have mainly focused on detecting pneumonia and pulmonary embolism on chest CT scans [10-15]. To our knowledge, no previous work has addressed the automated identification of acute heart failure on chest CT. This study introduces an algorithm capable of detecting all 3 conditions in the acute setting.

### Objectives

We hypothesize that AI algorithms can identify radiological conditions requiring urgent intervention with diagnostic accuracy comparable to current clinical radiology practice.

The aim of this protocol is to outline the framework for developing and validating AI algorithms to support this initiative. The proposed algorithms should be able to automatically analyze chest CT scans with the potential for providing immediate feedback on the likelihood of dyspnea-related diagnoses. The primary focus will be on cardiac decompensation, with the model also serving as a foundation for detecting pneumonia and pulmonary embolism.

## Methods

### Study Design

**Figure 1:**
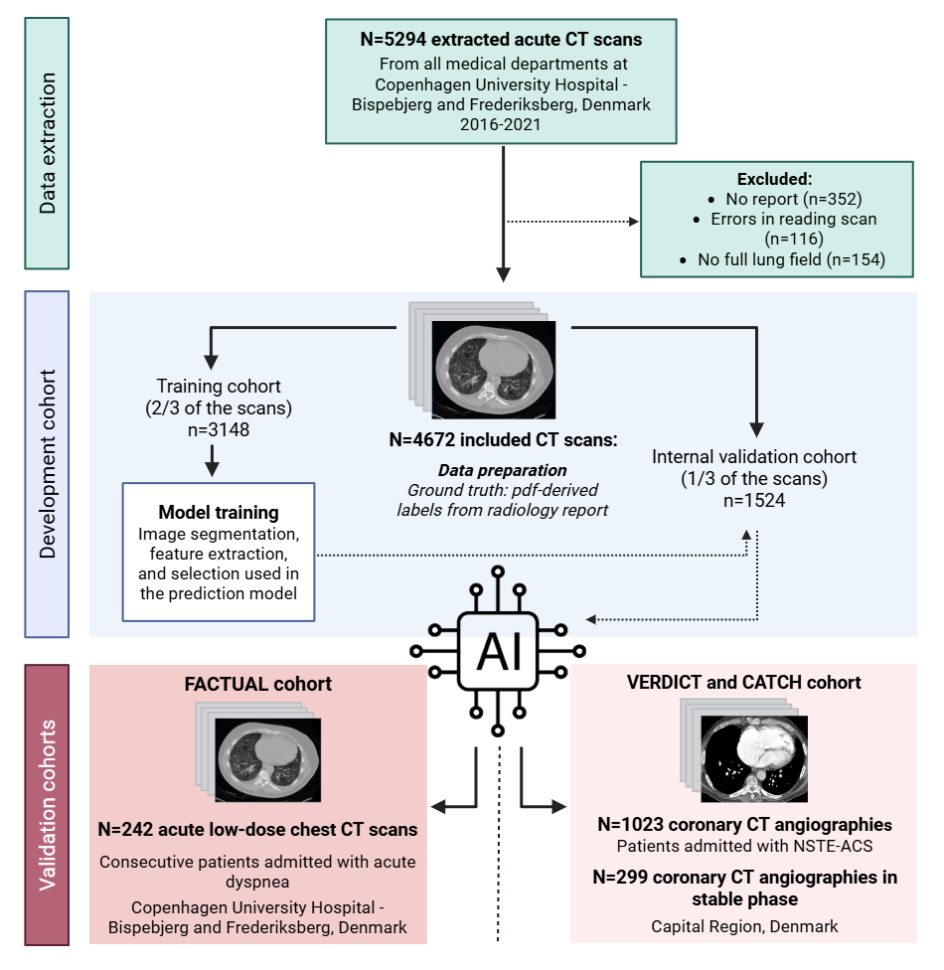
Overview of development and validation cohorts. We did not extract CT studies from participants from the FACTUAL study (year 2017-2019) or for patients below 18 years of age. CT: Computed Tomography; NSTE-ACS: non–ST-segment elevation acute coronary syndrome. Created with Biorender.

This is a retrospective study, including method development and validation (Figure 1).

### Training and Internal Validation Cohort

We extracted CT scans with accompanying radiological reports from all medical departments at the Copenhagen University Hospital–Bispebjerg and Frederiksberg Hospital, Denmark, conducted from January 1, 2016, to December 31, 2021. Consequently, 12,348 CT scans from 5111 studies of 4672 individual patients were extracted (Figure 1).

The scans originated from all medical wards ordering acute CT scans as standard diagnostic procedures. All types of CT scans (chest CT and abdominal scans, including the lungs) were included, encompassing various manufacturers, contrast-enhanced scans, noncontrast scans, low-dose protocols, and various imaging phases. Given the comprehensive inclusion of all acute chest CT examinations, the indications for these scans were broad and included assessments of chest pain, dyspnea, suspected pulmonary embolism, suspected pulmonary infection, suspected pneumothorax, or pleural effusion. We did not extract CT scans for patients aged 18 years or younger and participants in the Feasibility of Computer Tomography in Patients with Acute Decompensated Heart Failure (FACTUAL) study, as it served as a clinical validation cohort [7].

### Development of the AI Algorithm

We will develop an individual AI algorithm for automatic interpretation of radiologic signs of cardiac decompensation on chest CT scans, but the algorithm will also serve as a foundation for pneumonia and pulmonary embolism. The model will be trained to detect each condition, pulmonary congestion, pneumonia, and pulmonary embolism, independently, using a multilabel approach with separate binary classifiers. This allows more than one condition to be identified in the same scan. The CT scans were extracted in pseudonymized form.

Participants will be randomly split on the participant level into training (2/3) and internal test data (1/3; Figure 1). This process will be carried out separately for each of the 3 diseases, creating distinct AI models for each. We will train boosted tree prediction models using volume, diameter, and density measurements from structures segmented in the CT images. The segmentations will be derived from TotalSegmentator, extreme gradient boosting, and in-house codes [16,17].

We do not expect substantial missing data, as all CT scans will be extracted retrospectively without patient consent. Extreme, noisy, or missing feature values, typically caused by segmentation errors, will be handled by allowing them as many splits as possible in the tree-based model. To develop the model, a three-step procedure will be applied for boosted trees models: (1) parameter tuning with all features, (2) feature selection by forward selection guided by internal feature importance measures of extreme gradient boosting, and (3) retuning with the selected features. Hyperparameter optimization will be conducted by grid search using the area under the receiver operating characteristic curve as the evaluation metric. Parameter tuning and feature selection will be performed on the training data with stratified 3-fold cross validation, defined at the participant level and kept consistent across all steps.

We will use grid search and forward feature selection to find the best parameters. To further assess performance and enhance model interpretability, results will be presented using Shapley Additive Explanations (SHAP) [18]. The overall impact of each feature will be illustrated with SHAP beeswarm and bar plots. SHAP waterfall plots will explain the contribution of individual features to specific predictions, supported by visual inspection of the corresponding segmentations. This analysis allows for the removal of features deemed clinically unimportant or redundant. To evaluate the AI algorithms, they will be qualitatively evaluated by visual inspection, including segmentations. We will qualitatively evaluate the AI algorithms and model predictions using receiver operating curve (ROC) curves and confusion matrices of the thresholded predictions. False positives and false negatives will be evaluated by chief physicians with a special interest in thorax radiology.

### Ground Truth for Development and Validation of the AI Algorithm

The ground truth for the development and validation will be the radiological description of each CT scan, prepared by the on-call radiologist following standard clinical procedures. The on-call radiologists include both board-certified specialists and junior radiologists in training. All final assessments are supervised by a board-certified radiologist. All radiological reports are produced as part of routine clinical practice, and while variations in descriptions may occur due to specific clinical focuses of the scans, the regional standard reporting procedures ensure a consistent approach to assessing pathologies. These radiological findings will be extracted from the radiology reports (PDF) using a set of regular expressions for the selected diseases. The report clinical labels for cardiac decompensation may encompass terms such as pulmonary congestion, pulmonary edema, heart failure, and decompensation in the description of the radiologist. A subset of training data will be used to iteratively improve expressions. All reports containing these indicators, whether affirming or negating their presence, will be manually reviewed to correct any labeling errors. All labels will be reviewed for technical accuracy, with unclear cases assessed by project clinicians. False positives and negatives will be evaluated by chief physicians specializing in thoracic radiology.

Given the large volume of scans, manual review by an expert radiologist is impractical and resource intensive, but this approach maximizes the dataset, reflects real-world workflow, and mitigates risks to model validity.

### Target Condition Distribution

Previous studies from Denmark have reported that acute heart failure accounts for approximately 6% of medical hospital admissions among adults [19], that the incidence of pneumonia requiring hospitalization was 442 per 100,000 person years in 2003 [20], and that the incidence of pulmonary embolism requiring hospitalization occurs at a rate of 64 per 100,000 adults annually [21]. Preliminary labeling of the development dataset shows a similar distribution, with 7% to 8% positive for cardiac decompensation, while labeling for pneumonia and pulmonary embolism is still ongoing.

### Validation of the AI Algorithm

The AI algorithms will be internally validated using the area under the curve from ROC curves and confusion matrices. Optimal thresholds for the likelihood of heart and lung diseases will be obtained from the validation cohort and defined by a true positive rate and true negative rate of 90% or greater. These thresholds will be tested for the prediction of heart and lung diseases in the validation cohorts.

### Validation in Chest CT Scans in the Clinical FACTUAL Cohort

The AI algorithm for not only cardiac decompensation but also pneumonia will be validated in a previously performed clinical prospective observational study. The study included 242 consecutive patients with dyspnea, who underwent acute low-dose, noncontrast, chest CT scans within 12 hours from admission (FACTUAL study [H-17000869]; Textbox 1) [7]. CT scans were acquired without contrast at low dose (<2 mSv) using a multislice scanner and standard lung and soft tissue reconstruction kernels. A firewall ensured that none of these patients were included in the AI development sets.

**Inclusion and exclusion criteria for the FACTUAL, VERDICT (Very Early Versus Deferred Invasive Evaluation Using Computerized Tomography), and CATCH (Cardiac CT in the Treatment of Acute Chest Pain) studies.**

**Inclusion criteria for the FACTUAL study**

- Aged >50 years
- Acute dyspnea, combined with at least 1 abnormal objective parameter supporting respiratory imbalance
  Respiratory rate of more than 20 breaths per minute
  Saturation below 95% (if known chronic obstructive pulmonary disease (COPD) below 92%)
  Abnormal partial pressure of oxygen (pO2) or partial pressure of carbon dioxide (pCO2) in arterial blood
  Objective signs of heart failure (ie, jugular vein distention, peripheral edema, orthopnea, and bilateral rales on auscultation)
  Rhonchi or prolonged breathing on auscultation

**Exclusion criteria for the FACTUAL study (none of the exclusion criteria must be met)**

- All study examinations, including low-dose chest computed tomography (CT) scans, performed later than 12 hours after admission
- Patients who are unstable
  Requiring ventilation (including noninvasive ventilation)
  Requiring intensive care (inotropic treatment)
  Requiring telemetry due to acute coronary syndrome
- Patients who refuse or cannot provide consent (ie, mental illness [eg, dementia] and language barriers)
- Patients without Danish citizenship
- Life expectancy <3 months (assessed by the emergency department physician)
- CT thorax already indicated (pulmonary embolism, aortic dissection, or aneurysm)
- Patients who are isolated (ie, vancomycin-resistant *Escherichia coli* and influenza)

**Inclusion criteria for the VERDICT study**

- Invasive coronary angiography clinically indicated and logistically possible within 12 hours from the non–ST-segment elevation acute coronary syndrome (NSTE-ACS) diagnosis
- Aged >18 years
- Clinical suspicion of ACS, combined with at least one of the following high-risk criteria:
  Electrocardiogram (ECG) changes indicating new ischemia (new ST-segment depression, horizontal or downsloping ≥0.05 mV in 2 consecutive leads, and T-wave inversion >0.01 mV in 2 leads with prominent R-wave or R/S ratio >1)
  An increase in coronary markers of ischemia (troponin)

**Exclusion criteria for the VERDICT study (none of the exclusion criteria must be met)**

- Pregnancy
- Patients who refused or could not provide consent
- Indication for acute invasive coronary angiography (very high-risk NSTE-ACS)
- Life expectancy <1 year
- Known intolerance to platelet inhibitors, heparin, or x-ray contrast that could not be remedied medically
- Previous coronary artery bypass grafting
- Creatinine >140 μmol/L
- Known atrial fibrillation
- Women aged <45 years were not considered eligible for coronary CT angiography

**Inclusion criteria for the CATCH study**

- Hospitalized with suspicion of NSTE-ACS
- Normal or nondiagnostic ECG findings
- Normal troponins
- Discharged within 24 hours without recurrence of chest pain
- Clinical indication for further noninvasive, outpatient cardiac evaluation, evaluated by the treating physician

**Exclusion criteria for the CATCH study (none of the exclusion criteria must be met)**

- Aged <18 years
- New diagnostic ECG changes with ST-segment elevation or depression >0.5 mm or T-wave inversion >4 mm in ≥2 contiguous leads
- Women of childbearing age not using approved contraception
- Geographical residence or mental or physical conditions that could complicate follow-up
- Known allergy to iodinated contrast agents
- Serum creatinine >130 mg/L
- Abnormal chest radiography or blood test results that could explain chest pain
- Previous coronary artery bypass grafting
- Patients who refused or could not provide consent

The primary outcome is to validate the diagnosis of the AI algorithm in identifying the following: (1) normal CT images without treatment-requiring pulmonary changes, (2) radiological signs of cardiac decompensation, and (3) radiological infiltrates indicative of pneumonia.

The gold standard is the radiological evaluation of the CT images, which were analyzed in 2 ways:

1. Randomly analyzed by the on-call clinical radiologists according to clinical standards. The on-call radiologists had access to the medical journal, laboratory reports, and previous radiology images. The workflow is as follows: a radiology report is made by the on-call clinical radiologist and subsequently approved by a senior radiologist.
2. Two blinded, independent specialist thoracic radiologists evaluated every CT scan for distinct signs of heart and pulmonary diseases according to the Fleischner Society list [7,22].

### Validation in Coronary CT Angiograms in the VERDICT and the CATCH Studies

#### Overview

We aim to calibrate and validate the AI algorithm for cardiac decompensation on coronary CT angiographies (CCTA) in the Very Early Versus Deferred Invasive Evaluation Using Computerized Tomography (VERDICT) trial and in the Cardiac CT in the Treatment of Acute Chest Pain (CATCH) trial.

#### VERDICT Study

The VERDICT study was a large-scale randomized-controlled trial conducted in the Capital Region, Denmark, that included 2147 patients with non–ST-segment elevation acute coronary syndrome (NSTE-ACS). Patients were randomized to invasive coronary angiography within 12 hours or standard invasive care within 48 to 72 hours. In a substudy, 1023 participants from both groups underwent CCTA before the invasive procedure (Textbox 1) [23]. CCTA was performed on 64- or 320-detector scanners, predominantly with prospective electrocardiogram-triggering and intravenous contrast injection [23].

#### CATCH Study

In a randomized, controlled trial, 299 patients with acute chest pain but normal electrocardiogram findings and troponin values were randomized to CCTA in a stable phase and 301 to standard care (Textbox 1) [24]. CCTA was performed on a 320-detector scanner with standard premedication and contrast protocols [24].

The primary purpose is to investigate whether the AI-generated label of cardiac decompensation will have an independent prognostic value to predict death and heart failure hospitalization within 3 years of follow-up. An automatic AI report of cardiac decompensation on CCTA may guide early heart failure treatment for patients with NSTE-ACS. This is beneficial, as the lung field of CCTA often is underused but can provide important complementary information about pulmonary diseases.

### Ethical Considerations

This study is approved by the Danish National Committee on Health Research Ethics (1575037). All data used for algorithm development and validation were pseudonymized before research access.

The data were accessed for research purposes during the period from 2022 to 2023. We collected information on clinical indications for CT scans, radiographic diagnoses, and patient sex and age, with no other sensitive data obtained. Permission to use patient data was granted based on the determination that the study serves the public interest and only involves pseudonymized data. Therefore, the requirement for oral or written consent was waived. The validation cohorts were approved under separate ethical permissions and required written informed consent from all participants [7,23,24]. No compensation was provided to participants.

### Dissemination of Research Outcomes

Incidental findings are expected to be few due to the retrospective nature of the data collection as part of the clinical workflow; however, any findings identified during AI-assisted validation will be handled in accordance with the approved study protocol. Cases meeting predefined clinical significance criteria will be reviewed by a multidisciplinary safety committee and, if appropriate, communicated to the participant.

In terms of dissemination, the findings of this study are planned to be published in a high-impact, peer-reviewed journal and will be presented at international conferences, ensuring widespread dissemination and engagement with the broader scientific community (Multimedia Appendix 1). A separate technical methods article will be prepared and published independently. That methods article will provide a comprehensive and transparent description of the AI algorithm development, including data preprocessing, feature extraction, model training, and evaluation procedures (Multimedia Appendix 1).

### Data Management

All data are pseudonymized and stored securely on encrypted institutional servers with restricted access. Data extraction and coding are performed using automated scripts, with quality control checks applied during preprocessing.

### Statistical Analyses

In each validation cohort, the default thresholds obtained from the internal validation will be used to calculate the diagnostic ability of the AI algorithm to predict the prespecified heart and lung diseases. We will, for all diagnoses, calculate odds ratios, sensitivity, specificity, positive and negative predictive values, positive and negative likelihood ratios, and area under the curve from the ROC curves.

### Power Calculation

To account for variations in scanner generations, we included chest CT scans from all medical departments at a large hospital from 2016 onward, yielding approximately 5000 scans. Our initial estimation suggested the need for access to at least 15,000 scans to develop an algorithm with high diagnostic accuracy, as we expected that not all extracted scans would be suitable for algorithm development. Factors such as multiple scans per patient, incomplete thoracic coverage, and preprocessing challenges reduced the number of usable scans. Furthermore, we anticipated a distribution of diagnoses among patients with dyspnea, including heart failure, pneumonia, and chronic obstructive pulmonary disease exacerbations [9]. Given these considerations, we expect that our dataset of 5000 scans will provide a sufficient foundation for reliable training and validation of the AI model.

## Results

This is a retrospective study based on previously collected imaging data. As of August 2025, we have completed the extraction of CT scans and accompanying radiological reports. Natural language processing, CT segmentation, and the final stages of developing the AI algorithm are completed for pulmonary congestion but are still ongoing for the remaining conditions. The development and validation of the AI model constitute the main components of the study and are ongoing. Validation using 3 preexisting cohorts (FACTUAL, VERDICT, and CATCH) is planned (Multimedia Appendix 1). No new data collection or participant recruitment is planned, as all data have already been collected under previous ethics approvals.

A modified SPIRIT (Standard Protocol Items: Recommendations for Interventional Trials) schedule (Multimedia Appendix 1) outlines the timeline for key milestones, including algorithm development, internal validation, expert misclassification review, external validation in independent cohorts, and dissemination. We expect validation to conclude in late 2025, with final study results expected in 2026.

## Discussion

### Anticipated Findings

#### Overview

This study aims to develop and validate AI algorithms to automatically analyze chest CT scans and deliver instant feedback on the probability of dyspnea-related conditions. Our primary focus is on detecting cardiac decompensation while also laying the groundwork for assessing pneumonia and pulmonary embolism. To broadly test the algorithm, we evaluate the algorithm for cardiac decompensation in 3 different cohorts. The intended use of the algorithms is to assist in the detection of these conditions in a hospital setting, particularly in acutely admitted patients presenting with dyspnea. The algorithm is designed to support physicians, both radiologists and nonradiologists, by aiding in the early identification of critical findings while awaiting the final radiologic report. The AI model will provide interpretability through SHAP values for participant-level explanations, supported by visual inspection of segmentations and a numerical probability score for each detected disease. Its primary role will be to expedite early decision-making by highlighting relevant imaging features, potentially reducing time to diagnosis and treatment initiation. Optimizing the use of CT scans is crucial given their increasing prevalence. Currently, there is no standardized routine for describing cardiac decompensation on chest CT scans, presenting a gap that AI applications can fill.

#### AI Applications in Chest CT: Previous Evidence

CT scans are highly accurate for diagnosing cardiac decompensation and pulmonary diseases, often revealing subtle signs missed by chest radiographs [5,7]. Although CT scans involve higher radiation exposure, the advent of low-dose and ultra-low–dose chest CT scans has mitigated some of this concern while maintaining their diagnostic accuracy [25]. While it is unlikely to replace chest radiographs soon, CT imaging is frequently used when chest radiographs are inconclusive, especially in patients with undifferentiated dyspnea. It is important to address the upcoming challenges, including the shortage of radiologists and the need for AI assistance for nonradiologists. AI models can support physicians awaiting the final radiologic report and may alleviate radiologists’ workload, as AI models have been shown to reduce radiology reporting times by up to 22% [26].

To our knowledge, no published research has designed AI algorithms specifically to detect radiologic signs of cardiac decompensation on chest CT scans. One study demonstrated that machine learning–based quantification of lung water could identify increases in extracellular lung water in patients with heart failure with preserved ejection fraction, even in the absence of symptoms [27]. Previous research has highlighted the effectiveness of AI in diagnosing pulmonary conditions, including lung nodules [10], COVID-19, and COVID-19–related pneumonia [11,12]. A recent review emphasized that AI models can quantitatively assess pneumonia severity using CT images [13]. However, existing studies on CT imaging have primarily focused on patients with COVID-19. Our study extends this application, providing new insights into the use of AI for diagnosing different types of pneumonia. Limited research exists on AI and the detection of pulmonary embolism. One study demonstrated superior sensitivity and negative predictive values of AI models for pulmonary embolism detection, thereby reducing the risk of missed diagnoses [14]. Another study showed that an AI model aids detection and prioritization of incidental pulmonary embolisms on CT scans, but it was conducted in oncology patients [15].

Our study will assess the diagnostic efficacy of AI models in a broad population of hospital patients undergoing acute chest CT scans and across multiple diagnostic categories. For future clinical applications, the performance of the AI algorithms must be examined across various cohorts to demonstrate reliable differentiation between patients with and without critical conditions. At this stage, considerations such as diagnostic performance, regulatory approval, and integration into clinical workflow remain essential factors to establish the true clinical utility of these AI models.

#### Mitigating Imaging Variability and Validation Thresholds

Differences in imaging protocols across cohorts, including variations in scanner type, reconstruction kernels, and acquisition parameters, may affect AI model performance. To address this, the algorithm will be developed using data from multiple manufacturers, image types, and reconstruction kernels, and it will include both contrast-enhanced and noncontrast scans. This approach is designed to minimize the impact of technical variability and improve generalizability across different clinical settings, reducing the risk of bias from scanner settings and acquisition protocols. Planned cross-cohort validation will further ensure the robustness of the algorithm.

The sensitivity and specificity thresholds of 90% or greater chosen for validation are ambitious, but they align with performance levels generally regarded as necessary for clinical decision support in acute care, where the consequences of false negatives and false positives can be substantial. Thus, these thresholds serve as a target for ensuring patient safety and diagnostic reliability. We acknowledge that achieving such levels may involve trade-offs, including impaired sensitivity-specificity balance or reduced generalizability due to increased model complexity. High false positive rates could burden clinicians with unnecessary alerts, while false negatives might delay diagnosis of critical conditions.

### Strengths and Limitations

First, we use on-call radiological image descriptions as ground truth for developing and internally validating the AI algorithms. This is a limitation, as clinical reports may contain flaws or misdiagnoses, potentially compromising the accuracy of the AI algorithms. However, our approach allows for the algorithms to be easily trained on a large dataset and later be refined with higher-quality datasets as performance issues are identified in subsequent validations.

Second, the AI models are developed and will be validated on CT scans in the FACTUAL study, all obtained from the same university hospital in the Capital Region. While the diverse development cohort includes a broad range of acute chest CT scans, the FACTUAL cohort consists solely of low-dose, noncontrast chest CT scans from patients with dyspnea. To ensure independent validation in a clinical setting, FACTUAL data were strictly excluded from the development dataset. However, broader validation is necessary in diverse patient cohorts from other regions. We aim to extend validation to coronary CT angiography scans from the VERDICT and CATCH studies, which were acquired in different hospitals and time periods. However, these datasets lack radiological reports and primarily assess the algorithm’s prognostic value. The validation will provide evidence of the algorithm’s applicability in external cohorts, but we also underscore the need for further external validation using datasets with radiological ground truth.

Third, differences in imaging protocols across cohorts, such as variations in scanner type, reconstruction kernels, and acquisition parameters, may influence AI model performance in external cohorts. To mitigate these effects, the algorithm will be calibrated using normalization, data augmentation, and cross-cohort validation to ensure robustness. Such strategies are intended to minimize the impact of technical variability and enhance generalizability across different clinical settings, but site-specific biases need to be addressed in future cohorts.

Fourth, although the AI model will be trained on well-characterized cohorts, its future performance may be affected by variations in demographic, technical, and clinical factors outside the study setting. Demographic differences, such as age, sex, and comorbidities, could influence the appearance of the disease on CT scans. Technical factors, including scanner models, acquisition parameters, and image reconstruction methods, may alter feature representation and introduce site-specific biases. Clinical workflow differences, such as referral patterns or labeling practices, could further affect the distribution and definition of target conditions. These challenges are inherent to all nonmulticenter studies. Consequently, the algorithm will likely require further adaptation and validation before being deployed commercially or applied globally. To mitigate these issues, this protocol emphasizes standardized labeling procedures and the inclusion of diverse training data with multiple scanning protocols and scanner brands. In addition, external validation across diverse populations and imaging settings is needed to further strengthen the model.

Successful clinical implementation will also require regulatory approval, user-friendly integration, and appropriate clinician engagement. If the developed AI model demonstrates satisfactory performance in the planned validation cohorts, subsequent work will examine how best to integrate it into routine clinical workflows; however, this is not encompassed by this protocol. Finally, potential risks of AI, including clinician overreliance and false negative results, must be carefully considered. AI outputs are intended to support, not replace, clinical judgment.

A strength of this study is that the AI algorithms will be developed in a cohort of undifferentiated patients from all medical departments ordering acute scans. We included diverse CT images conducted with different scan protocols, reconstruction kernels, and scanner models and vendors, both contrast and no contrast and low dose and high dose. The FACTUAL patient cohort for the validation is well validated, with comprehensive and detailed radiologic labels provided by 2 blinded thoracic expert radiologists.

### Conclusions

In conclusion, this study will develop AI algorithms that enable automatic analyses of acute CT scans in adult patients for the detection of acute heart and lung diseases across several populations of patients who are acutely hospitalized. This work is a critical first step, establishing a methodology that can guide subsequent multicenter and international validation studies.

## Appendix

Multimedia Appendix 1 Planned SPIRIT (Standard Protocol Items: Recommendations for Interventional Trials) study schedule.

## Abbreviations

AI: artificial intelligence
CATCH trial: Cardiac CT in the Treatment of Acute Chest Pain
CCTA: coronary computed tomography angiography
CT: computed tomography
FACTUAL trial: Feasibility of Computer Tomography in Patients with Acute Decompensated Heart Failure
NSTE-ACS: non−ST−segment elevation acute coronary syndrome
ROC: receiver operating curve
SHAP: Shapley Additive Explanations
SPIRIT: Standard Protocol Items: Recommendations for Interventional Trials
VERDICT trial: Very Early Versus Deferred Invasive Evaluation Using Computerized Tomography

## Data Availability

Data sharing is not applicable to this article as no new datasets were generated or analyzed during this study protocol. The TotalSegmentator software and the boosted trees model implementation are publicly available. Upon publication of the study results, the source code for the AI algorithm will be made available in a public repository (GitHub).
