# Supplementary material for: AI-Based Algorithm to Detect Heart and Lung Disease From Acute Chest Computed Tomography Scans: Protocol for an Algorithm Development and Validation Study
Source: JMIR Res Protoc. 2025 Sep 19;14:e77030. doi: 10.2196/77030 (PMC12495367; doi:10.2196/77030)
Supplement: Multimedia Appendix 1 [file resprot_v14i1e77030_app1.docx]

**Multimedia Appendix 1**

Planned SPIRIT (Standard Protocol Items: Recommendations for Interventional Trials) study schedule.

| Time point | 2022-2023 | 2024 | 2025 (quarters 1-4) | | | | 2026 (quarters 1-4) | | | |
| --- | --- | --- | --- | --- | --- | --- | --- | --- | --- | --- |
| Data collection | Active | |  |  |  |  |  |  |  |  |
| Extraction of CT^a^ scans | ✓ |  |  |  |  |  |  |  |  |  |
| Radiological report extraction | ✓ |  |  |  |  |  |  |  |  |  |
| Natural language processing | ✓ |  |  |  |  |  |  |  |  |  |
| CT segmentation |  | ✓ |  |  |  |  |  |  |  |  |
| AI^b^ algorithm development |  | Active | | |  |  |  |  |  |  |
| Training of model |  | Active | | |  |  |  |  |  |  |
| Internal validation |  | Active | | |  |  |  |  |  |  |
| Expert clinical evaluation of misclassifications |  |  |  | ✓ |  |  |  |  |  |  |
| Validation |  |  | Active | | | | | |  |  |
| FACTUAL^c^ cohort |  |  | Active | | |  |  |  |  |  |
| CATCH^d^ and VERDICT^e^ cohorts |  |  |  |  |  | Active | | |  |  |
| Dissemination (submission) |  |  |  |  |  |  |  |  |  |  |
| Protocol article |  |  |  | ✓ |  |  |  |  |  |  |
| Development and method article |  |  |  |  | ✓ |  |  |  |  |  |
| FACTUAL validation |  |  |  |  |  | ✓ |  |  |  |  |
| CATCH and VERDICT validation |  |  |  |  |  |  |  |  | ✓ |  |

^a^CT: computed tomography.

^b^AI: artificial intelligence.

^c^FACTUAL: Feasibility of Computer Tomography in Patients with Acute Decompensated Heart Failure

^d^CATCH: Cardiac CT in the Treatment of Acute Chest Pain

^e^VERDICT: Very Early Versus Deferred Invasive Evaluation Using Computerized Tomography
